# Tropical Fruits and Their Co-Products as Bioactive Compounds and Their Health Effects: A Review

**DOI:** 10.3390/foods10081952

**Published:** 2021-08-22

**Authors:** Sonia Sayago-Ayerdi, Diana Laura García-Martínez, Ailin Cecilia Ramírez-Castillo, Heidi Rubí Ramírez-Concepción, Manuel Viuda-Martos

**Affiliations:** 1 Tecnologico Nacional de Mexico, Instituto Tecnologico de Tepic, Av Tecnológico 2595, Col Lagos del Country, Tepic 63175, Nayarit Mexico, Mexico; ssayago@tepic.tecnm.mx (S.S.-A.); dilagarciama@ittepic.edu.mx (D.L.G.-M.); aceramirezca@ittepic.edu.mx (A.C.R.-C.); heruramirezco@ittepic.edu.mx (H.R.R.-C.); 2 IPOA Research Group, Centro de Investigación e Innovación Agroalimentaria y Agroambiental (CIAGRO-UMH), Agro-Food Technology Department, Miguel Hernández University, Orihuela, 03312 Alicante, Spain

**Keywords:** flavonoids, phenolic acids, tropical fruits, health, bioactive compounds, anti-carcinogenesis, anti-inflammatory, cardiovascular disease

## Abstract

Tropical and subtropical fruits are recognized as a source of a high content of bioactive compounds and health promoting properties due to their nutritional composition. These beneficial health effects are related to the content of several of these bioactive compounds, mainly flavonoids and non-flavonoid phenolics. Many of these compounds are common in different tropical fruits, such as epicatechin in mango, pineapple, and banana, or catechin in pineapple, cocoa or avocado. Many studies of tropical fruits had been carried out, but in this work an examination is made in the current literature of the flavonoids and non-flavonoid phenolics content of some tropical fruits and their coproducts, comparing the content in the same units, as well as examining the role that these compounds play in health benefits.

## 1. Introduction

Tropical fruits are a botanically varied group of fruits originally from tropical and sub-tropical zones. Most of these fruits are comestible and their consumption is growing in both national and international markets owing to the rising recognition of their nutritional and health-promoting properties as well as their organoleptic characteristics including their exclusive taste, sensory properties, and mouthfeel [1]. According to their crop extension, production volumes, and market, tropical fruits may be classified as principal, secondary and wild fruits [2]. The principal fruits include mango (*Mangifera indica* L.), pineapple (*Ananas comosus* L. Merr), avocado (*Persea americana* Mill.), papaya (*Carica papaya* L.), and banana (*Musa paradisiaca* L.), among others. Secondary and wild fruits include guava (*Psidium guajava* L.), tamarind (*Tamarindus indica* L.), açai (*Euterpe oleracea* Mart.), purple passion fruit (*Passiflora edulis* Sims), pomegranate (*Punica granatum* L.), coconut (*Cocos nucifera* Linn.), date (*Phoenix dactylifera* L.), cacao (*Theobroma cacao* L.), acerola (*Malpighia emarginata* D.C.), rambutan (*Nephelium lappaceum* L.), lychee (*Litchi chinensis* Sonn.), noni (*Morinda citrifolia* L.), camu-camu (*Myrciaria dubia* McVaugh), dragon fruit (*Hylocereus* spp.), mangosteen (*Garcinia mangostana* L.), goldenberry (*Physalis peruviana* L.), etc. [3,4].

Therefore, in recent years, the production and commercialization of this type of fruit have increased significantly, estimating an annual growth of around 3.8% [5]. Approximately, 99% of tropical fruit production comes from low-income developing countries mainly in Latin America and Caribbean regions. Brazil, Colombia, and Ecuador are the main producers and exporters of tropical fresh fruit worldwide, although there is also a small production from Asiatic and African areas [6]. About 50% of the tropical and subtropical fruit production is intended for the fresh fruit market, and 50% is used in numerous processed forms (desserts, nectars, compotes, marmalades, sauces, syrups, snacks, jellies, flours, and wines), the most important being tropical fruit beverages [7]. Nutrient-rich juices obtained from tropical fruits are gaining acceptance owing to the desire of consumers for healthy products whilst experiencing new and exotic flavors, and at the same time due to the focus of industry on producing beverages with health claims as a parameter of differentiation [8]. This fact is also influenced by the growing interest on the part of consumers in how fruits and their derivatives can help maintain health, and the role that diet plays in the prevention and treatment of many diseases [9].

**Figure 1:**
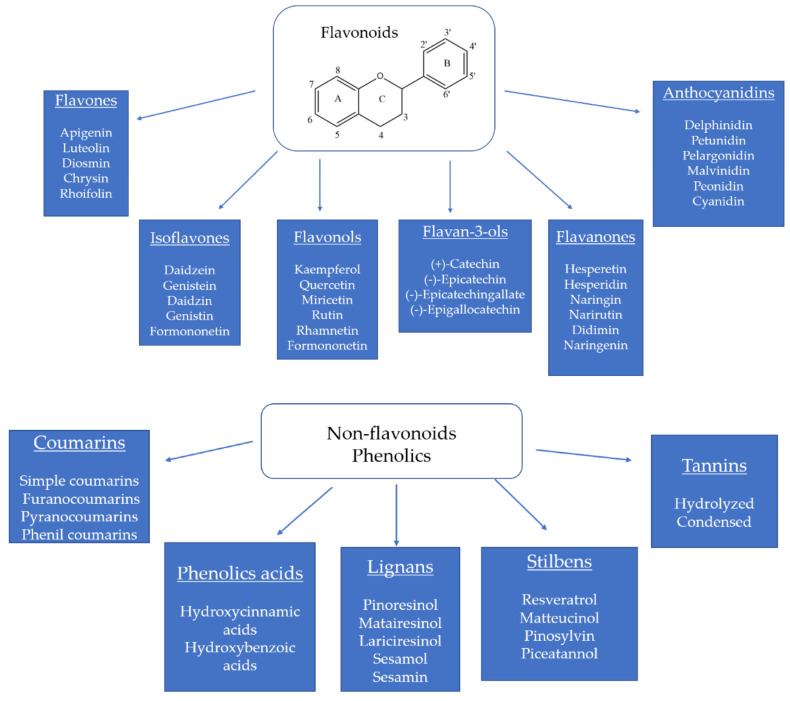
Flavonoid family and non-flavonoid phenolics found in tropical fruits and their coproducts [25,26,27,28,29,30,31,32,33,34,35,36].

Beyond their macro- and micronutrient contents, the edible and non-edible parts of tropical fruits are an important source of numerous bioactive compounds including carotenoids, sterols and stanols, phenolic compounds such as flavonoids and non-flavonoids phenolics [6,10,11], and the dietary fiber content. In recent years, the flavonoids and non-flavonoids phenolics, in all their types and structures (Figure 1), obtained from tropical fruits and their coproducts have aroused the interest of different fields, such as biomedical science and food technology. This interest in these bioactive compounds is due to their numerous biological effects including antioxidant [12,13], hypo-cholesterolemic [14,15], anti-cancer [16,17], immunomodulatory [18], anti-inflammatory [19,20], anti-diabetic [21,22] and anti-hypertensive [23,24], among others.

This work reviews the currently available literature concerning flavonoid and non-flavonoid phenolics composition of several tropical fruits and their coproducts, as well as the health benefits of their consumption, aiming to provide information for additional studies, as well as to promote the consumption and cultivation of these fruits.

## 2. Flavonoids and Non-Flavonoid Phenolics in Tropical Fruits

**Figure 2:**
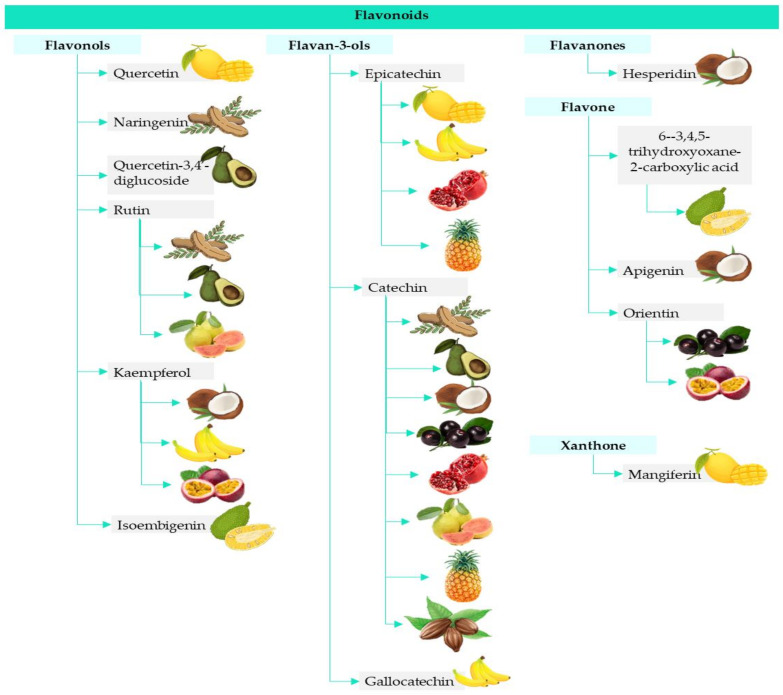
The main flavonoids found in tropical fruits [25,26,27,28,29,30,31,32,33,34,35,36].

Flavonoids and non-flavonoids phenolic compounds are one of the main classes of plant secondary metabolites, with numerous different compounds identified (among them over 8000 flavonoids) [11]. This class of bioactive compound displays a huge variety of structures, including monomeric, dimeric, and polymeric phenols, which are responsible for their bioactive properties [37]. As mentioned previously, the edible and non-edible parts of tropical fruits are an important source of this type of bioactive compound (Figure 2). However, as can be seen throughout this review, it is very difficult to obtain a harmonization of results. This is because each researcher, laboratory or even journal in which the results are published uses a different method of obtaining the extracts, analyzing them and expressing them. Often, when researchers are looking for information about, tropical fruits and their coproducts the number and characteristics of different compounds identified are the same, but in this document we focus on flavonoids that have been quantified and identified in some tropical fruits, so that the reader can see in an easy way which are common. Certainly, this point of view is a new outlook on the relevance and importance of these compounds, along with which are present and in what amount. Regarding this final point, all the values used have been expressed in the same units to express a clearer vision of the composition of this type of bioactive compound.

### 2.1. Açai

The predominant bioactive constituents of açai fruits (*Euterpe oleracea* Mart.) are flavonoids, especially anthocyanins, and flavanones. Xiong et al. [25] reported that the principal anthocyanins found in industrially processed açai pulp were cyanindin-3-*O*-rutinoside (values ranged between 0.29 and 2.18 mg/g), cyanindin-3-*O*-glucoside (0.31–1.08 mg/g), and peonidin-3-*O*-glucoside (0.04–0.10 mg/g). Previously, Garzón et al. [38] reported that the main flavonoids found in Colombia açais fruits were the flavones orientin (0.15 mg/g), isovitexin (0.12 mg/g), homoorientin (0.10 mg/g) and vitexin (0.10 mg/g) whilst the principal anthocyanins were cyanidin-3-*O*-glucoside and cyanidin-3-*O*-rutinoside. Crespo-López et al. [39] reported that the main flavonoids found in açai juice were cyanindin-3-*O*-rutinoside (448.05 mg/L), orientin (381.02 mg/L) and taxifolin deoxyhexose (308.00 mg/L). Açaí seeds, a co-product of açai juice extraction, have been reported to have a high content of flavan-3-ols, mainly catechin (15.67 mg/g), epicatechin (5.32 mg/g), and their polymers, procyanidins B1 and B2, with values of 16.08 and 1.49 mg/g, respectively [40].

### 2.2. Pomegranate

In pomegranate (*Punica granatum* L.) it is possible to find numerous types of phytochemicals including flavonoids such as anthocyanins, including cyanidin-3-O-glucoside (22.77 mg/g), cyanidin-3,5-*O*-diglucoside (25.36 mg/g) and pelargonidin-3,5-*O*-diglucoside 11.16 mg/g) [26], flavons and flavonols (kaempferol, luteolin, quercetin, rutin, naringin) and flavan-3-ols (catechin, epicatechin, gallo-catechin) [41], and condensed and hydrolysable tannins including punicalagin with values ranging between 87.8 and 453 mg/g, and punicalin [42] and non-flavonoid phenolics such as ellagic acid, caffeic acid, ferulic acid, chlorogenic acid, and gallic acid, among others [43]. As regards anthocyanin content, this are mainly concentrated in the arils and, therefore, in the juice. In this sense, Díaz-Mula et al. [44] reported that total anthocyanins (Cyanidin-3,5-*O*-diglucoside, delphinidin-3,5-*O*-diglucoside, pelargonidin-3-*O*-glucoside, delphinidin-3-glucoside, and cyanidin-3-glucoside) content found in arils was 1.63 mg/g, while in pomegranate juice this was 2.03 g/L. The peel is composed basically of non-flavonoid phenolics. Thus, El-Hadary and Ramadan [14] reported that the main non-flavonoid phenolics identified in pomegranate peel were puicalagin (98.00 mg/g), followed by ellagic acid (12.5 mg/g) and gallic acid (2.50 mg/g).

### 2.3. Passion Fruit

Regarding the flavonoid and non-flavonoid phenolics content of yellow passion fruit (*Passiflora edulis* Sims f. *Flavicarpa*), several studies have described the presence of C-glyco-sylflavones as the main flavonoids found in the peel of these fruits, schaftoside, iso-schaftoside, iso-orientin, orientin, isovitexin, vitexin, luteolin-6-C-chinovoside and luteolin-6-C-fucoside, and vicenin-2 being the main constituents [27,45]. On the other hand, in the pulp of passion fruit it is possible to find flavonols and flavan-3-oles. Thus, Carmona-Hernandez et al. [46] reported that (+)-catechin; (−)-epicatechin, and quercetin 3-glucoside were the main constituents of yellow passion fruit lyophilized pulp cultivated in Colombia. A previous study by dos Reis et al. [47] reported that the major flavonoids present in the pulp of yellow passion fruit cultivated in Brazil were quercetin (5.06 mg/g) and kaempferol (1.99 mg/g).

### 2.4. Guava

Guava fruit (*Psidium guajava* L.) contains high amounts of phytochemicals, mainly flavonoids and phenolic acids. Nunes et al. [28] reported that fresh red guava cultivated in Brazil had a high content of the flavonoids rutin and naringenin with values of 0.36 and 0.18 mg/g dry weight, respectively. In addition, they reported that 2-hydroxybenzoic acid and 3,4-dihydroxyphenylacetic acid were the main non-flavonoid phenolics, with values of 0.11 and 0.22 mg/g, respectively. Lopes dos Santos et al. [48] reported that rutin and catechin, with values of 0.24 and 0.02 mg/g, were the principal flavonoid found in white guava, whilst the highest concentrations found for phenolic acids were ellagic acid (0.15 mg/g), gallic acid (0.07 mg/g) and vanillic acid (0.02 mg/g). Da Silva Lima [49] reported that the main flavonoids and non-flavonoid phenolics found in guava pulp were quercetin (0.19 mg/g), myricetin (0.11 mg/g), and pinocembrin (0.11 mg/g), while the principal non-flavonoid phenolics were ellagic acid and gallic acid with values of 7.54 and 4.15 mg/g, respectively. More recently, Tan et al. [50] carried out a study to determine the polyphenolic profile of white and red guava fruits cultivated in China. These authors found that in both white and red guava the main flavonoids were procyanidin B1 (0.41 and 0.11 mg/g, respectively) and phloretin (0.12 and 0.11 mg/g, respectively). For non-flavonoid compounds, in both white and red guava crypto-chlorogenic acid and chlorogenic acid were predominant, with values of 0.07 and 0.05 mg/g and 0.04 and 0.08 mg/g, respectively.

### 2.5. Pineapple

Pineapple (*Ananas comosus* L. Merr.) is a tropical fruit widely cultivated in South America, which may be considered as one of the most valuable fruits for high value-added compounds due to the fact that, in their composition, is possible to find several bioactive compounds including dietary fiber, organic acids, bromelain, and mainly phenolic compounds and flavonoids. Sun et al. [29] analyzed the polyphenolic profile of 11 pineapple cultivars and found that the pineapple samples analyzed had several flavonoid compounds, among which catechin, with values ranging between 0.05 and 0.28 mg/g fresh weight, and epicatechin, with values between 0.01 and 0.02 mg/g fresh weight, were predominant. As regards the non-flavonoid phenolics, sinapinic and chlorogenic acids were the main components. Dominguez et al. [51] reported that the principal flavonoids found in the pulp of pineapple cv Esmeralda were catechin and epicatechin with values of 0.17 and 0.05 mg/g dry weight, while the main non-flavonoid phenolics were gallic acid (0.97 mg/g) and vanillic acid (0.02 mg/g). Similarly, Arampath and Dekker [52] reported that the main flavonoids found in pineapple pulp cultivated in Sri Lanka were catechin and epicatechin with values of 0.64 and 0.38 mg/g fresh weight.

### 2.6. Mango

Mango (*Mangifera indica* L.) stands out for its functional value; it generally contains polyphenolic compounds such as mangiferin (0.26 mg/g), phenolic acids such as sinapic acid (0.01 mg/g) and gallic acid (0.02 mg/g), benzophenones and flavonoids such as quercetin (0.01 mg/g) [53,54]. Nevertheless, significant differences in flavonoids and non-flavonoid phenolic compounds between varieties of mango have been reported, and the unripe fruit extract are more abundant in these compounds than ripe fruits [30]. The main phenolic compounds in mango pulp include mangiferin, gallic acid, catechins, quercetin, kaempferol, protocatechuic acid, ellagic acids, propyl and methyl gallate, rhamnetin, and anthocyanins [55]. Mango peel is a rich source of bioactive compounds such as polyphenols. In this way, Nguyen et al. [56] established that mango peel mainly contained galloyl glucoside, mangiferin and quercetin hexoside. Similarly, Sáyago-Ayerdi et al. [57] detected gallo-tannins from 5 (penta-*O*-galloyl glucose) to 13 galloyl units (trideca-*O*-galloyl glucose). The flavonoids dominant in mango kernel were mangiferin, homo-mangiferin, iso-mangiferin, anthocyanins, kaempferol, and quercetin, while gallic, ellagic, 4-caffeoylquinic acids, caffeic, coumaric, protocatechuic, and ferulic are the main non-flavonoid phenolics found in mango kernel [58,59].

### 2.7. Avocado

Avocado (*Persea americana* Mill.) shows a high content of flavonoid and non-phenolic compounds which can be found in pulp, seed, or peel [60]. Araujo et al. [61] identified 15 major compounds in the peel, such as 11 procyanidins, dimers and trimers in different isomer shapes. Trujillo-Mayol et al. [31] identified that the main flavonoids present in avocado peel were epicatechin (0.02 mg/g) and hesperidin (0.01 mg/g). In addition, there were non-flavonoid phenolics such as caffeic acid (0.2 mg/g), coumaric acid (0.1 mg/g) and ferulic acid (0.01 mg/g) [62]. The avocado seed has also shown the presence of flavonoids (catechin 1.02 mg/g dry weight) and other non-flavonoid phenolics including chlorogenic acid, caffeic acid and ferulic acid with values of 0.75, 0.22 and 0.01 mg/g dry weight, respectively [63]. Other authors reported 11 compounds in avocado seed, tyrosol glucoside (2.23 mg/g), 1-caffeoylquinic acid (1.12 mg/g), and hydroxy-tyrosol glucoside (0.39 mg/g) being the major compounds obtained [64], whereas the avocado pulp contained phenolic compounds such as *p*-coumaric acid glucoside (0.08 mg/g), 5-feruloylquinic acid (0.05 mg/g) and *p*-coumaric acid (0.04 mg/g), among others [60,64]. 

### 2.8. Tamarind

Tamarind (*Tamarindus indica* L.) is a fruit rich in phytochemicals, mainly polyphenols, flavonoids, alkaloids, triterpenes and polysaccharides, responsible for the healing properties of the plant [32,65]. The presence of polyphenols in tamarind is dominated by pro-anthocyanidin groups such as pro-cyanidin B2, apigenin, catechin, epicatechin, pro-cyanidin dimers, and trimers, eriodyctiol, taxifoline, and naringenin [32,66,67,68], catechin (27.00 mg/g), gallic acid (0.93 mg/g) and naringenin (0.50 mg/g) being most dominant [69,70]. The seed, considered as waste, also has a wide range of flavonoid compounds. It is a rich source of naringenin (95.30 mg/g), catechin (54.93 mg/g) and rutine (15.36 mg/g). It is also possible to find several non-flavonoid phenolics such as gallic acid (2.14 mg/g), caffeic acid (4.35 mg/g), and *p*-coumaric acid (2.09 mg/g) [70].

### 2.9. Coconut

Edible parts and various value-added products of coconut (*Cocos nucifera* Linn.) contain considerable amount of phenolics, among them flavonoid compounds with biological activity potential. Arivalagan et al. [33] reported the presence of 28 phenolic compounds in coconut tests, including 16 non-flavonoids (phenolic acids) and twelve flavonoids. No-flavonoid compounds were mainly phenolic acids such as trans-cinnamic acid (1.06 mg/g), protocatechuic acid (0.87 mg/g), *p-*coumaric acid (0.25 mg/g), or *o*-coumaric acid (0.21 mg/g), among others such as chlorogenic acid, vanillic acid, syringic acid, ferulic acid, caffeic acid, and gallic acid. The flavonoids found were catechin, luteolin, rutin, myricetin, quercetin, naringenin, epicatechin, and epigallocatechin, the main components being apigenin (0.38 mg/g), hesperidin (0.43 mg/g), and kaempferol (3.09 mg/g). Mahayothee et al. [71] determined, in matured coconut meat, the presence of salicylic acid, *p*-hydroxybenzoic acid, syringic acid, m-coumaric acid, *p*-coumaric acid, gallic acid, caffeic acid, and catechin as its main phenolic compounds. Coconut shell fibers also have polyphenolic compounds including gallic acid (4.30 mg/g), syringic acid (0.42 mg/g), catechin (0.49 mg/g), and epicatechin (0.42 mg/g) [72]. Virgin coconut oil contains non-flavonoid compounds such as phenolic acids; the phenolic acids present are protocatechuic, vanillic, caffeic, syringic, ferulic, and *p*-coumaric acids [73].

### 2.10. Cocoa

Cocoa (*Theobroma cacao* L.) has been proposed as a rich source of bioactive compounds including flavonoids and non-flavonoid phenolics. The main flavonoid present in all cacao structures is flavan-3-ol. Therefore, Peláez et al. [74] studied the flavonoid profile present in cocoa beans and reported a catechin contents of 0.65 mg/g for fresh cocoa beans and 0.53, 0.27, and 0.16 mg/g for beans fermented for 48, 96, and 120 h, respectively. Recently, Urbańska et al. [75] reported that the principal flavonoids found in unroasted cocoa beans were epicatechin (2.61 mg/g), procyanidin B2 (1.07 mg/g), and (+)-catechin (0.13 mg/g). Cocoa bean shell, the main co-product from the cocoa industry, is also a great source of bioactive compounds, thus Botella-Martinez et al. [34] reported that catechin and epicatechin were the main flavonoids found in this co-product with values of 4.56 and 6.34 mg/g dry weight, respectively. Similarly, Hernández-Hernández et al. [76] reported that epicatechin (values ranging between 4.40 and 34.97 mg/g) and catechin (0.55 to 3.33 mg/g) were the main flavonoids of cocoa bean shells obtained from different genotypes of cacao cultivated in Mexico.

### 2.11. Banana

Banana (*Musa paradisiaca* L.) has a potential health value because of its chemical content. The peel constitutes approximately 40% of the weight of the banana and has the highest concentration of flavonoids. Currently, more than 40 polyphenolic compounds have been identified, flavonoids being the most predominant forms, highlighting gallic acid (3.86 mg/g), catechins, epicatechins (1.14 mg/g), gallo-catechins (5.91 mg/g), and anthocyanins [77,78]. Anyasi et al. [35] reported that epicatechin, gallo-catechin and myricetin-3-*O*-ramnosyl-glycoside are the flavonoids with the highest concentration in ripe bananas.

### 2.12. Jackfruit

Jackfruit (*Artocarpus heterophyllus* L.) has different structural parts, the peel, pulp, axis, flake, seed coat, and seed kernel [36], but each part of the fruit contains different phenolic compounds. Li et al. [79] found jackfruit axis extract contained principally three phenolics (elenaic acid, Urolithin D, 3-(6-hydroxy-7-methoxy-2H-1,3-benzodioxol-5-yl) prop-2-enoic acid) and one flavonoid (iso-embigenin); Wang et al. [36] found in the leaves of Jackfruit five flavones including artocarpin, cudraflavone C, albanin A, cudraflavone, and brosimone I, two flavanones, norartocarpanone and euchrenone a7, six 2-arylbenzofuran derivatives, moracin M, moracin C, albafuran B, artoindonesianin B-1, demethylmoracin I, and moracin D, one stilbenoid, artocarbene, one lignan, 2,6,2′,6′-tetramethoxy-4,4′-bis(2,3-epoxy-1-hydroxy-propyl)biphenyl and one triterpenoid, griffithine A. Likewise, Fang et al. [80] reported, in fruit and roots, three phenolic compounds, namely artocarpesin, norartocarpetin, and oxy-resveratrol.

### 2.13. Other Fruits

Table 1 shows the flavonoid and non-flavonoid phenolics found in different parts of several tropical fruits.

## 3. Biological Effects

Tropical fruit consumption has demonstrated numerous beneficial health effects (Table 2) which are related to the high content of bioactive compounds present in these fruits. Beneficial health properties of bioactive compounds include antioxidant, antiproliferative, anti-inflammatory, neuroprotective, antihypertensive, hypo-cholesterolemic, and hypoglycemic properties [17,66,70,80]. These beneficial effects can be used to prevent several diseases including cardiovascular, cancer, and diabetes mellitus diseases as well as neurodegenerative diseases [96,97,98,99,100,101,102,103,104,105,106,107,108,109,110,111,112,113,114,115,116,117,118,119,120,121]. Table 2 shows several biological effects of flavonoids and non-flavonoid phenolics present in tropical fruits. These theoretical approaches and hypotheses on the potential role of plant secondary metabolites such as flavonoids and non-flavonoids phenolics against several diseases seem to be reasonable, as most of them have been justified so far, either with in vitro or in vivo studies or by different interventional clinical trials in humans.

From our point of view, a greater number of studies with humans has to be carried out to ensure, in a conclusive way, all the potential benefits provided by the consumption of fruits in general and tropical fruits in particular, which provide a large number of bioactive compounds.

### 3.1. Açai

Açai (*Euterpe oleracea* Mart.) is a berry-like fruit native to the Amazon region which has recently gained popularity in North America and Europe due to its bioactivity demonstrated in in vitro and in vivo studies, including anti-inflammatory, antioxidant, antimicrobial, antinociceptive, anticancer, anti-atherogenic, and healing activities [122,123]. Several of these compounds have been associated with preventing the development of several chronic diseases. For instance, some research studies have been published reporting the consumption of açai berries, açai juice or açai extracts with the potential to prevent dyslipidemia, or as chemoprotective agents against cancer [107,124,125,126]. In this respect, the effect on the blood lipid profile of consumption of 200 mL/day of açaí juice during four weeks with a 4-week washout period was analyzed by de Liz et al., [124]. These authors reported that, after four weeks, açaí juice increased the concentrations of high-density lipoprotein cholesterol by 7.7%. Additionally, açaí juice intake improved the oxidative stress biomarkers significantly, and increase in total antioxidant capacity, catalase, and glutathione peroxidase by 66.7%, 275.1%, and 15.3%, respectively, while the oxidative stress index decrease was 55.7% compared to baseline. Previously, Pala et al., [126] reported that açaí pulp consumption (0.2 kg/day for 28 days) had no effect on blood lipid profile parameters of healthy women. However, açaí pulp intake increased the serum levels of apolipoprotein A1 and the activity of paraoxonase-1, whose molecules are high-density lipoprotein cholesterol precursors.

Another very important health property of açai fruit berry is its anti-cancerogenic activity. Therefore, some studies have inquired about the antitumoral properties of açai. For example, Barros et al. [102] analyzed the cytotoxicity of açai seeds extract against several tumor cell lines including MCF-7 (breast adenocarcinoma), NCI-H460 (non-small cell lung cancer), HeLa (cervical carcinoma) and HepG2 (hepatocellular carcinoma). According to the authors, açaí seeds extract, with a high content of flavan-3-ols (i.e., catechins and procyanidins), had an inhibitory effect against tumor cell lines assayed, being more effective for the cervical carcinoma cell line with a GI50 of 18 μg/mL. Choi et al. [127] reported that the consumption of açai berries (pellets containing 5%) decreased the incidence of both adenoma (from 76.9% to 23.1%) and cancer (from 76.9% to 15.4%), as well as the expression of proinflammatory cytokines, including tumor necrosis factor α, interleukin-1β and interleukin-6, in colorectal cancer. Fragoso et al. [107] carried out a study to assess the potential protective effects of açaí pulp in a colitis-associated carcinogenesis rat model. These authors found that ingestion of lyophilized açai pulp with a high concentration of anthocyanins (mainly cyanidin glycosides) decreases the total number of aberrant crypt foci, aberrant crypt foci multiplicity, tumor cell proliferation and incidence of tumors with high-grade dysplasia. In addition, açai pulp enhanced the gene expression of negative regulators of cell proliferation, as well as inflammation. In the same way, Martins et al. [128] found that the viability of the human lung carcinoma cell line (A549) decreases (72.07%) with açai extract (200 µg/mL) treatment after 48 h. In addition, açai extracts increased the percentage of cells in the G0/G1 phase and promoted a high growth of apoptotic cells, compared with non-treated cells.

### 3.2. Pomegranate

Pomegranate (*Punica granatum* L.) fruits are consumed worldwide, fresh, or processed in the form of juice, jam, wine, oil obtained from seeds and in extract supplements [129]. In the composition of both peel and arils, it is possible to find numerous types of phytochemical including flavonoids and non-flavonoid phenolics. Due to this high content of these bioactive compounds an extensive range of health benefits have been attributed, including anti-inflammatory, antioxidant, antibacterial, and antiviral properties, as well as playing a role in lipid regulation and immunomodulation [41,130,131].

As mentioned above, pomegranate and pomegranate derivates have demonstrated several health benefits. In this sense, Sohrab et al. [132] carried out a study to investigate the effects of pomegranate juice consumption (200 mL/day for 6 weeks) on the blood lipid profile of healthy volunteers. These authors reported that, at the conclusion of the intervention, total cholesterol, total triglycerides, low-density lipoprotein cholesterol and high-density lipoprotein cholesterol did not show any change between the intervention group and the control group, but total cholesterol and low-density lipoprotein cholesterol decreased significantly compared to pre-trial values within the intervention group. El-Hadary and Ramadan [14] conducted a study to analyze the oral administration of pomegranate extract at 200 mg/kg for 56 days on hyperlipidemic rats. According to the authors significantly lower values were achieved for total lipid, total cholesterol, low-density lipoprotein cholesterol and very low-density lipoprotein cholesterol, while high-density lipoprotein cholesterol levels increased. In a similar study, Grabež et al. [130] evaluated the effects of pomegranate peel extract consumption on plasma lipid profile and fatty acid level in patients with diabetes mellitus type 2. These authors reported that plasma levels of triglycerides, low-density lipoprotein cholesterol/high-density lipoprotein cholesterol ratio, and HbA1c were significantly reduced, whilst the level of high-density lipoprotein cholesterol was augmented, compared to placebo intake. 

In reference to the preventive effects of pomegranate on carcinogenesis, Pan et al. [96] investigated the effect of punicalagin, one of the most important bioactive compounds found in pomegranate, on the cellular process in breast cancer and its molecular mechanism. These authors found that a high dose of punicalagin (50 μM or higher) inhibited the invasion potential, migration, and viability of breast cancer cells. In addition, the expression of N-Cadherin, Golgi phosphoprotein 3, matrix metalloproteinase-2, and matrix metalloproteinase-9 decreased. Similarly, Nasser et al. [133] investigated the antitumor effect of pomegranate juice on human lung adenocarcinoma basal epithelial cells (A549). These authors reported that the treatment of A549 cells with pomegranate juice, which had a high content in flavonoids and non-flavonoid phenolic compounds, produced a decrease in the viability of these cells in a concentration-dependent manner. Thus, for lyophilized pomegranate juice concentrations of 300 and 600 µg/mL a reduction of 26% and 49% of cell viability, respectively, was achieved.

Another interesting property of pomegranates is their anti-inflammatory activity. Therefore, the anti-inflammatory activity of pomegranate extracts, or pomegranate juice had been described by numerous in vitro and in vivo studies. Karwasra et al. [134] analyzed the anti-inflammatory activity of pomegranate rind extracts. They reported that pomegranate extracts at 200 mg/kg reduced pain and inflammation by downregulating the activation of tumor necrosis factor R1, tumor necrosis factor alpha, interleukin-1β, interleukin-6, nuclear factor-κB, oxidative stress markers, and tissue histology. In a previous study, Stojanović et al. [135] reported that a pomegranate peel extract rich in punicalin, punicalagin, and ellagic acid improved the nitric oxide levels in peritoneal macrophages and attenuated the levels of interleukin-17 and interferon-gamma in mesenteric lymph node cells obtained from mice with type 1 diabetes.

### 3.3. Passion Fruit

The fruits of the genus *Passiflora* showed a high content of bioactive compounds, mainly polyphenolic compounds, which have demonstrated several health benefits such as anti-inflammatory activity, anti-cancerogenic properties, and the prevention of cardiovascular diseases [136,137]. Regarding the latter, Barbalho et al. [136] studied the daily ingestion of passion fruit juice on the biochemical profile of diabetic rats. These authors found that, at the end of the study (30 days), the rats showed significantly reduced total cholesterol, triglyceride, and low-density lipoprotein cholesterol levels, and an increased high-density lipoprotein cholesterol level. Fernandes Marques et al. [138] analyzed the effects of daily ingestion of passion fruit peel flour in patients with HIV. The authors reported that, at the end of the study (90 days), this (30 g) was effective in reducing total cholesterol (15.1% with respect to the initial value) and triglycerides (16.1% with respect to the initial value) after 30 days. The concentrations of low-density lipoprotein cholesterol decreased by 12.7% with respect to initial values, while high-density lipoprotein cholesterol increased by 13.4% with respect to the initial value.

Another important property of *Passiflora* spp. is its anti-cancer activities. Thus, Ballesteros-Vivas [137] analyzed the anti-proliferative potential of *Passiflora mollissima* Bailey against HT-29 colon cancer cells. These authors reported that, after treatment for 48 and 72 h, the viability of HT-29 colon cancer cells was evidently affected, while low effects were obtained on normal human colon fibroblast cells. The bioactive extract was shown to block HT-29 cells in the S and G2/M phases of the cell cycle, which could be facilitated by the inactivation of the FAT10 cancer signaling pathway. Kasala et al. [139] analyzed the anticancer effect of chrysin, a flavonoid found in fruits of the *Passiflora* genus. The authors reported that chrysin treatment (250 mg/kg body weight) in mice significantly reduces the benzo(a)pyrene-induced lung carcinogens. Thus, chrysin supplementation downregulated the expression of proliferating cell nuclear antigen, cyclooxygenase-2 and nuclear factor-κB, where these proteins maintained cellular homeostasis.

### 3.4. Guava

Guava (*Psidium guajava* L.) is a popular fruit of tropical and subtropical countries. In its composition, it is possible to find several bioactive compounds These bioactive nutrients play significant roles in several health problems related to lifestyle diseases, such as diabetes (type 2), cardiovascular disease, several cancer types and obesity. As regards the hypo-cholesterolemic properties of guava, Maryanto and Marsono, [140] carried out a study to assess the potential of red guava in reducing cholesterol content in hyper-cholesterolemic rats. They found that red guava intake significantly reduced the contents of total cholesterol (32%), low-density lipoprotein cholesterol (43%), and triglyceride (18%). On the other hand, red guava intake significantly increased high-density lipoprotein cholesterol content (18%). Shabbir et al. [141] mentioned that diet supplementation with polyphenols, obtained from guava pulp, significantly increased the level of high-density lipoprotein in groups fed with polyphenols compared to control groups. Additionally, total cholesterol and low-density lipoprotein levels were significantly decreased.

Another very important health-related property of guava is its anticarcinogenic activity. Therefore, Feng et al. [142] analyzed the cytotoxic activities of several flavonoids (guavinosides and quercetin glycosides) obtained from ethanolic extract of guava fruits. These authors reported that the guavinosides and quercetin glycosides inhibited the cell proliferation from gastric carcinoma cells SGC-790, A-549, and HeLa cells. Recently, in an interesting study, Correa et al. [143] evaluated the anticarcinogenic potential of three guava cultivar fruit pulp extracts in MDA-MB-435 and MCF-7 human breast cancer cells. The author reported that, after 48 h of treatment, all the guava cultivars’ extracts caused reduction of MDA-MB-435 and MCF-7 cell viability. Additionally, all guava extracts caused MDA-MB-435 and MCF-7 cell count reduction in G0/G1 and G2/M phases and increased apoptosis. Guava fruits also showed anti-inflammatory properties. Thus, Li et al. [144] reported that the expression of inflammatory proteins, such as iNOS and NF-κB, was suppressed via activated PPARγ, and the expression levels of GPx3 and ACO increased in rats fed with guava fruit for 8 weeks.

### 3.5. Pineapple

Pineapple (*Ananas comosus* L. Merr), based on the content of its bioactive compounds, could be considered a fruit with a high number of health benefits. Thus, Saxena and Panjwani [145] reported that treating rats with hydroalcoholic extract of pineapple (200–400 mg/kg/day, oral) for a period of 30 days reduced the values of cholesterol, low-density lipoprotein, very low-density lipoprotein and triglycerides, whilst it increased high-density lipoprotein and total protein levels. Similarly, Seenak et al. [146] reported that daily pineapple ingestion reduced serum lipid profiles, atherogenic coefficient, cardiac risk ratio, and liver enzyme activity. Additionally, daily ingestion of pineapple also restored cardiac protein carbonyl content, and decreased the cardiac malondialdehyde and the cardiac pro-inflammation cytokine Interleukin-6 and Interleukin-1β levels.

### 3.6. Mango

Mango (*Mangifera indica* L.) is the world’s most commonly produced tropical fruit, and is originally from Asia [147,148]. As mentioned above, this fruit showed multiple biological effects due to its high content of bioactive compounds. Therefore, the most active biological constituent of mango is the xanthone known as mangiferin, with biological properties such as antiviral, anticancer, antidiabetic, antioxidative, antiaging, immunomodulatory, hepatoprotective and analgesic effects, and also as a promising therapeutic agent against neurodegenerative disorders [116,149].

Flavonoids and non-flavonoid phenolics present in mango have multiple biological effects. These phytochemicals have shown high antioxidant, nutraceutical, antimicrobial, anticancer, and pharmaceutical significance and antiproliferative activities [59]. Worth noting mangiferin, as one of the major compounds in mango, may be a compound with high cancer chemo-preventive potential and possible preventive effect against oncogenesis [150]. In this sense, Gold-Smith et al. [115] confirmed that mangiferin causes a decrease in the cell cycle by detecting an abnormal alteration, thereby reducing proliferation, metastasis and promoting apoptosis of malignant cells.

Potential health benefits have also been studies, including antibiotic capacity, prebiotic effects, and their effect in non-communicable chronic diseases such as obesity, diabetes mellitus, cancer, cardiovascular, neurological, and inflammatory diseases [151].

### 3.7. Avocado

Avocado (*Persea americana* Mill.) is an important native fruit of Mexico and Central America, cultivated in tropical and subtropical regions of the world. This fruit is also considered very valuable because it has a complete nutritional content associated with many health benefits due to the high levels of unsaturated fatty acids, minerals, fiber, proteins, vitamins and phenolic compounds [102,152]. This high content of phenolic compounds confers to avocado numerous biological activities, including antifungal, antibacterial, antidiabetic, antihypertensive, anti-inflammatory, anticancer, and hypo-cholesterolemic properties [31,61,103,153,154]. Ervianingsih et al. [155] used avocado seed waste in biscuits as an alternative food for people with diabetes, because studies demonstrated that the flavonoids could reduce blood glucose levels through glucose absorption or by increasing insulin secretion, can stimulate glucose absorption in peripheral tissues, and regulate the work of enzymes involved in carbohydrate metabolic pathways.

Tremocoldi et al. [103] determined that epicatechin and catechin had antioxidant activity since they can stabilize peroxyl radicals, superoxide radical and hypochlorous reactive species. Likewise, Dabas et al. [17] found that avocado seed extract has antioxidant capacity and significant bioactivity in reducing cell viability and inducing apoptosis in human prostate, colon, lung and human breast cancer cells. The presence of hydroxycinnamic acid derivatives in avocado has an important antioxidant effect and anti-inflammatory properties. Alam et al. [156] suggest that these compounds may enhance cardiovascular health by exerting blood pressure-lowering effects and by acutely improving the endothelial function. There is also an effect on adipose tissues, inhibiting macrophage infiltration and nuclear factor κB (NF-κB) activation in obese animals.

The presence of procyanidins in avocado has been involved in different beneficial effects. Eldaim et al. [157] reported that procyanidins mitigate DNA damage in tumoral kidneys through a mechanism that involves P53 ANDPCNA proteins expression, and normalize serum levels of urea, creatinine, potassium, and chloride. Enayati et al. [108] found, in an in vivo model, that procyanidins exert cardioprotective effects through a mechanism mediated via the release of nitric oxide, increasing the antioxidant activity and scavenging of reactive oxygen species. Likewise, the procyanidins have a potentiating effect, both in vitro and in vivo, on a broad range of antibiotic classes against pathogenic *Escherichia coli, Proteus mirabilis, and Pseudomonas aeruginosa*. Evidence that this acts by repressing two antibiotic resistance mechanisms, selective membrane permeability, and multidrug efflux pumps, has been presented [158,159].

Other studies show that catechin compounds quercetin, and hesperidin, among other flavonoids, have the potential to be used in the design of coadjutant therapies against viral infection [160]. Russo et al. [161] found flavonoids such as quercetin, baicalin, luteolin, hesperetin, gallo-catechin gallate, and epigallocatechin gallate interfere with the replication cycle of the coronavirus in vitro, therefore flavonoids and their derivative may represent target compounds to be tested in future clinical trials to enrich the drug arsenal against coronavirus infections. Wu et al. [162] reported that avocado extract can inhibit dengue virus replication via regulation of NF-κB-dependent induction of the antiviral interferon response. Finally, avocados do not contain potentially toxic or harmful compounds, making them a promising natural source of bioactive compounds for many applications in the food and pharmaceutical industries.

### 3.8. Tamarind

*Tamarindus indica* L., commonly known as tamarind, is a tropical fruit from Africa, India, and Thailand that belongs to the subfamily *Caesalpinioideae* of the family Leguminosae (Fabaceae) [32]. It is generally consumed fresh for its attractive bittersweet flavour and sometimes dried or ground in products such as juices, syrup, pulps, dehydrated fruits, gelatin, and sweet products [163,164]. Tamarind leaves, pulp, seeds, veins, and skins are sources of bioactive compounds with antioxidant, hypolipidemic, antihyperglycemic, antimicrobial, anti-inflammatory, analgesic, hepatogenic, antihypertensive, and spasmolytic properties [66,68,69,163,164,165]. The seed, considered as waste, also has a wide range of biological effects. This coproduct showed antioxidant capacity due to the inhibition of enzymes such as aldose, reductase, ATPase, lipoxygenase, and cyclooxygenase enzymes responsible for the generation of cytotoxic free radicals [166].

In an in vitro study conducted by Bhadoriya et al. [101], it was reported that consumption of tamarind seed extract (100 mg/kg at 250 mg/kg) may have a protective effect on pancreatic β-cells, increased glucose uptake, and decreased body weight, along with recovery of altered hematological parameters.

### 3.9. Coconut

The phenolic compounds of coconut (*Cocos nucifera* Linn.) have other health benefits. For example, the presence of flavonoids (quercetin and catechin) in the ethanolic extract of coconut husk fiber has photoprotective action. In addition, it contains antioxidant and antiglycation properties that can reduce the effects of oxidative damage caused by ultraviolet radiation, offering stability to sunscreens and assisting in the preservation of collagen [77]. Another biological activity of coconut is its implication in the prevention of microvascular diabetic complications. Sheela et al. [109] in an in silico analysis, reported that a coconut extract reduced the expression of aldose reductase, an enzyme implicated in the activation of the polyol pathway, responsible for triggering diabetic complications. A study carried out by Nikooei et al. [167] found that intake of 30 mL of virgin coconut oil, for four weeks in subjects with metabolic syndrome improves serum levels of triglyceride, high-density lipoprotein cholesterol, and fasting blood glucose.

### 3.10. Banana

Phenolic compounds are found in banana (*Musa paradisiaca* L.) in two forms; soluble conjugated and insoluble, covalently bound to sugars or cell wall components [168]. As the most important fraction are bound phenols rather than free ones, most of the reactions of these compounds begin when they reach the colon, where they act as chelating agents of the function of free radicals ROS and RNS, conferring the functional group in their nucleus structure [169]. Flavonoids can also inhibit enzymes, such as oxygenases (prostaglandin synthase), required in the synthesis of eicosanoids and capable of preventing the spread of bacterial and tumor metastases. Current studies have shown the ability of banana flavonoids to inhibit the main enzymes involved in cancer initiation and progression by preventing the production of nitric oxide and nuclear factor-kB [77]. It can be said that the banana, mainly the husk, is a potential source of bioactive compounds such as flavonoids and polyphenols, with a wide range of medicinal properties, in particular the high uptake of free radicals.

### 3.11. Cocoa

Cocoa (*Theobroma cacao* L.), due to the high content of bioactive compounds, mainly flavonoids and phenolic acids, has demonstrated several health properties including hypolipidemic, antihyperglycemic, anticancerogenic, anti-inflammatory properties, etc. In reference to hypolipidemic properties, Sansone et al. [170] reported that daily intake of 450 mg of cocoa flavonoids for 1 month decreases total cholesterol by 0.20 mmol/L and low-density lipoprotein cholesterol by 0.17 mmol/L, whereas high-density lipoprotein cholesterol increased by 0.10 mmol/L. Lin et al. [171] in a meta-analysis of 1131 participants reported that cocoa flavanol intake enhanced the biomarkers of lipid metabolism. Thus, the triglyceride levels decreased while high-density lipoprotein cholesterol increased. On the other hand, no significant changes were found for low-density lipoprotein cholesterol and total cholesterol. More recently, Davinelli et al. [172] reported that the consumption of 220 mg of cocoa flavonoids for 4 weeks causes a reduction in total cholesterol (−12.37 mg/dL), triglycerides (−3.81 mg/dL), and plasma low-density lipoprotein cholesterol (−14.98 mg/dL), compared with baseline. Additionally, they found that plasma high-density lipoprotein increased (+3.37 mg/dL) compared with baseline. 

Cocoa flavonoids have also demonstrated several anti-inflammatory properties. Thus, Álvarez-Cilleros et al. [173] reported that cocoa attenuates the levels of phospho-p65-nuclear factor-kappaB and the expression of inflammatory factors including intercellular adhesion molecule-1 (ICAM-1), vascular adhesion molecule-1 (VCAM-1) and inducible nitric oxide synthase in the aortas of rats. Rossin et al. [10] reported that ethanolic extracts (rich in epicatechin and tannins) obtained from cocoa bean shell had a positive effect on the prevention of oxysterol mixture-induced InterLeukin-8 release (pro-inflammatory cytokine) on Caco-2 intestinal cell models and the prevention of exaggerated toll-like receptor 2 and 4 (TRL2 and TRL4) responses, which may contribute to oxysterol-dependent intestinal inflammation.

### 3.12. Jackfruit

The different parts of Jackfruit (*Artocarpus heterophyllus* L.) contain different amounts and types of phenolic compounds. Therefore, Jackfruit has proved to be a natural source of multiple flavonoids with potent biological activities, including antioxidant, anti-inflammatory, antibacterial, antineoplastic, etc., but further research is warranted to explore the health benefits and specific compounds for their better utilization.

The anti-inflammatory effect of jackfruit was analyzed by Fang et al. [79]. These authors indicated that artocarpesin (flavonoid compound) suppressed the LPS-induced production of nitric oxide (NO) and prostaglandin E2 (PGE2) through the down-regulation of inducible nitric oxide synthase (iNOS) and cyclooxygenase 2 (COX-2) protein expressions. This study was determined the inhibitory effects on the production of proinflammatory mediators in lipopolysaccharide (LPS)-activated RAW 264.7 murine macrophage cells. Jackfruit extract also had an anticancer effect. In this sense, Li et al., [77] found that the extract showed excellent antioxidant activities in HepG2 cells, liver cell models for studying diseases related to this organ.

Flavonoids have been widely associated with a wide range of biological activities, highlighting their antimicrobial activity. The possible mechanism of action of flavonoids, mainly baicalein, was demonstrated by Yang et al., [99] who reported that this occurred through the inhibition of outflow pumps which decreased their efficiency, eventually leading to cell death or apoptosis. These pumps are the major cause of resistance by microorganisms to chemical synthesis fungicides. A study characterized and analyzed the antifungal activity of jackfruit leaf extract obtained using conventional and emerging technologies and found that a concentration of 5 mg/mL showed the highest percentage of inhibition against mycelial growth of *Penicillium itallicum* [174].

## 4. Conclusions

Tropical and subtropical fruits may be considered as a very important source of nutrients and bioactive compounds, mainly flavonoids and non-flavonoid phenolics. In this way, the consumption of this type of fruit should be recommended by several health organizations around the world for its beneficial health properties. In the same way, it is important to increase awareness of the beneficial health effect of the bioactive compounds that can be obtained by tropical fruits consumption. However, apart from the indisputable benefit of consuming fruits in general and topical fruits in particular, some not so beneficial aspects have to be taken into account that are related to the sustainability of the crops, the use of pesticides for a more widespread and better production, transport costs and the environmental consequences that these entailm as well as a greater awareness in relation to fair trade.

On the other hand, several investigations around the world need to be carried out in future with the aim of focusing on the bio-accessibility and bioavailability of bioactive compounds, mainly flavonoids and non-flavonoid phenolics present in these fruits. The clarification of the several variations and alterations that tropical fruit components might suffer during the gastrointestinal digestion could provide important information which may be applied in the development of novel food, new functional food or nutraceutical products.

## Figures and Tables

**Table 1:** Flavonoid and non-flavonoid phenolic compounds found in edible and non-edible parts of several tropical fruits.

Fruit	Source	Flavonoids	Non-Flavonoids Phenolics	Ref.
*Kiwi* *(Actinidia arguta (Siebold & Zucc.) Planch. ex Miq)*	Pulp	Rutin (0.33 mg/g dw) and kaempherol-3-*O*-galactoside (0.25 mg/g dw)	Crypto-chlorogenic acid (0.10 mg/g dw) and neochlorogenic acid (0.08 mg/g dw)	[81]
Acerola (*Malpighia emarginata* D.C.)	Pulp	Keampferol-3-*O*-glucoside (0.33 mg/g dw) and isorhamnetin (0.11 mg/g dw)	*p*-coumaric acid (0.29 mg/g dw)	[82]
Acerola (*Malpighia emarginata* D.C.)	Pulp and peel	Myricetin (0.94 mg/g dw) and rutin (0.61 mg/g dw)	2,5-dihydroxybenzoic acid (7.37 mg/g dw) and 3,4-dihydroxybenzoic acid (0.98 mg/g dw)	[83]
Jabuticaba (*Myrciaria jaboticaba* (Vell.) O.Berg)	Peel	Cyanidin-3-*O*-glucoside (7.25 mg/g dw) and quercetin-3-*O*-rutinoside (1.17 mg/g dw)	Ellagic acid (0.23 mg/g dw) and gallic acid (0.13 mg/g dw)	[84]
Jabuticaba (*Myrciaria jaboticaba* (Vell.) O.Berg)	Seeds	Castalagin (0.13 mg/g dw) and vescalagin (0.69 mg/g dw)	Ellagic acid (3.80 mg/g dw) and gallic acid (2.30 mg/g dw)	[85]
Rambutan (*Nephelium lappaceum* L.)	Seeds	Geraniin (0.42 mg/g dw) and corilagin (0.09 mg/g dw)	Ellagic acid (0.46 mg/g dw) and gallic (0.10 mg/g dw)	[86]
Lychee (*Litchi chinensis* Sonn)	Seeds	Rutin (0.098 mg/100 g dw) and scopoletin (0.076 mg/100 g dw)	N.D.	[87]
Dragon fruit (*Hylocereus* spp.)	Pulp	Myricetin (0.18 mg/g dw) and quercetin (0.06 mg/g dw)	Gallic acid (0.23 mg/g dw)	[88]
Kaki (*Dyospiros kaki* L.)	Pulp	Epicatechin (0.03 mg/g dw) and catechin (0.02 mg/g dw)	Gallic acid (2.96 mg/g dw)	[89]
Longan (*Dimocarpus longan* Lour)	Peel	Corilagin (2.15 mg/g dw)	Ellagic acid (0.08 mg/g dw) and gallic acid (0.18 mg/g dw)	[90]
Longan (*Dimocarpus longan* Lour)	Seed coat	Corilagin (5.53 mg/g dw)	Ellagic acid (0.83 mg/g dw) and gallic (0.49 mg/g dw)	[91]
Camu-camu (*Myrciaria dubia* McVaugh)	Seed coat	Rutin (0.13 mg/g dw)	Ferulic acid (0.59 mg/g dw) and *p*-coumaric acid (0.49 mg/g dw)	[92]
Camu-camu (*Myrciaria dubia* McVaugh)	Pulp	Catechin (0.12 mg/g dw) and myricetin (0.02 mg/g dw)	Ellagic acid (0.19 mg/g dw) and syringic acid (0.07 mg/g dw)	[93]
Noni (*Morinda citrifolia* L.)	Pulp	Catechin (1.11 mg/g dw), rutin (0.40 mg/g dw) and quercetin (0.27 mg/g dw)	Gallic acid (0.24 mg/g dw)	[94]
Noni (*Morinda citrifolia* L.)	Pulp	Scopoletin (0.53 mg/g dw) and rutin (0.54 mg/g dw)	N.D.	[95]

N.D.: No determined; dw: dry weight.

**Table 2:** Beneficial health effects of flavonoid and non-flavonoid phenolic compounds found in edible and non-edible parts of several tropical fruits.

Polyphenolic Compound	Biological Activity	Mechanism	Ref.
**Tannin**			
Punicalagin	Anticancer	Inhibition of the invasion, migration, and viability of breast cancer cells. Decreases the expression of N-Cadherin, Golgi phosphoprotein 3, matrix metalloproteinase-2, matrix metalloproteinase-9	[97]
**Flavonols**			
Quercetin-3-*O*-glucuronide	Anti-inflammatory	Decreases in ROS-associated inflammation by inhibition of interleukin-6 and tumor necrosis factor-α production with suppression of IKKβ/NF-κB phosphorylation.	[98]
	Antidiabetic	Facilitates the PI3K signaling by positive regulation of serine/tyrosine phosphorylation of insulin receptor substrate-1 (IRS-1).	
Quercetin	Photoprotective	Absorbs UV radiation, inhibits UV-induced inflammation in primary keratinocytes, and inhibits skin damage produced by UVB rays.	[99]
Naringenin	Antioxidative	Protects ocular neurons from degeneration, reverses retinal pigment epithelium degeneration and laser-induced choroidal neovascularization	[100]
**Flavone**			
Artocarpesin	Anti-inflammatory	Suppresses the LPS-induced production of nitric oxide (NO) and prostaglandin E2	[80]
Baicalein	Antifungal	Inhibition of outflow pumps, since they decreased their efficiency, which eventually led to cell death or apoptosis.	[100]
**Flavan-3-ols**			
Benzo-tropolona	Anticancer	Reduces the viability of human breast (MCF7), lung (H1299), colon (HT29), and prostate (LNCaP) cancer cells in vitro. After 12 h treatment on a model in vivo the G0/G1 phase cell cycle arrests in LNCaP cells.	[17]
	Antioxidant	Reduces lipid hydroperoxide formation in an oil-in-water emulsion (33% reduction at 500 μg/mL).	[17]
	Antiangiogenic	Inhibition of kinase proteins.	[70]
Catechin	Antidiabetic	Protective effect on pancreatic β cells Increased glucose absorption. Reduces the blood glucose level.	[101]
Catechins and procyanidins	Antitumor	Inhibitory effect against cervical carcinoma cell	[102]
Catechin and epicatechin	Antioxidant	Stabilizes peroxyl radicals, superoxide radical and hypochlorous reactive species.	[103]
Epicatechin	Antiproliferative capacity	Immunosuppressive effect on T cell proliferation	[104]
	Anti-inflammatory	Prevention of oxysterol mixture-induced InterLeukin-8 release (pro-inflammatory cytokine)	[105]
Gallo-catechin	Antioxidant	DPPH radical scavenging activity	[106]
**Anthocyanins**			
Cyanidin glycosides	Anticancer	Decreases the total number of aberrant crypt foci, aberrant crypt foci multiplicity, tumor cell proliferation and incidence of tumors with high grade dysplasia	[107]
**Pro-anthocyanins**			
Resveratrol	Cardioprotective	Effects via nitric oxide release, Nrf2 pathway, and antioxidant activity.	[108]
Procyanidin B2	Anti-obesity	Reduction of lipid accumulation in a dose dependent-manner. Hypolipidemic activity.	[66]
	Antioxidant	Scavenges radicals and inhibits xanthine oxidase.	[66]
**Hydroxycinnamic acids**			
Caffeic acid	Antidiabetic	Prevents diabetic complications specifically inhibiting aldose reductase dependent polyol pathway.	[109]
Ferulic acid	Neurological	Interactions with fibrils Aβ predominantly by hydrogen bonding with His14 and Glu22, interfering with the formation of β-sheets, thereby inhibiting the formation of aggregates.	[110]
**Hydroxybenzoic acids**			
Gallic acid	Chemo-preventive	Inhibition of activation of nuclear factor-Band Akt signaling pathways along with the activity of COX, ribonucleotide reductase and GSH. Activates ATM kinase signaling pathways to prevent the processes of carcinogenesis	[111]
	Anti-obesity	Inhibition of myeloperoxidase release and activity, scavenging of superoxide anions, as well as a possible interference with the assembly of active NADPH-oxidase, may account for the inhibition of the inflammatory process.	[112]
	Anti-inflammatory	Inhibition of myeloperoxidase activity, hence directly inhibiting the production of hypochlorous acid, and scavenging the hazardous reactive species produced by the enzyme.	[113]
	Antiallergic	Blocks the release of histamine, which would otherwise result in immediate hypersensitivity.	[114]
Ellagic acid	Anti-inflammatory	Improves the nitric oxide levels in peritoneal macrophages and attenuate the levels of interleukin-17 and interferon gamma in mesenteric lymph node cells obtained from mice with type 1 diabetes	[115]
**Xanthone**			
Mangiferin	Chemo-preventive	Decreases in the cell cycle by reducing proliferation, metastasis and promoting apoptosis of malignant cells.	[116]
	Neuroprotective	Up-regulates dopamine concentrations and diminishes neurotoxin’s effects which involve oxidative stress, mitochondrial dysfunction, and apoptosis.	[117]
		Improved object recognition memory in healthy rats through a mechanism that might involve an increase in neurotrophin and cytokine levels.	[118]
	Cardioprotective	Increases the concentration of phospholipids in cardiac tissue and restore the activity of antioxidant enzymes: superoxide dismutase, catalase, glutathione peroxidase, glutathione transferase and glutathione reductase.	[119]
	Anti-inflammatory	Inhibits the enzyme phospholipase A2, in human synovial secretion, and suppresses the release of inflammatory mediators prostaglandin E_2_ and leukotriene B4 by macrophages.	[120]
	Antidiabetic	Reduction of hyperglycemia and atherogenicity in diabetic rats through a significant decrease in blood glucose levels as well as a decrease in serum concentrations of triglycerides, total cholesterol and low-density lipoprotein cholesterol.	[121]

## Data Availability

The data presented in this study are available on request from the corresponding author.
